# Ethylcellulose–A Pharmaceutical Excipient with Multidirectional Application in Drug Dosage Forms Development

**DOI:** 10.3390/ma12203386

**Published:** 2019-10-17

**Authors:** Katarzyna Wasilewska, Katarzyna Winnicka

**Affiliations:** Department of Pharmaceutical Technology, Medical University of Bialystok, Mickiewicza 2c, 15-222 Bialystok, Poland; katarzyna.wasilewska@umb.edu.pl

**Keywords:** ethylcellulose, polymeric material, cellulose derivative, pharmaceutical excipient

## Abstract

Polymers constitute the most important group of excipients utilized in modern pharmaceutical technology, playing an essential role in the development of drug dosage forms. Synthetic, semisynthetic, and natural polymeric materials offer opportunities to overcome different formulative challenges and to design novel dosage forms for controlled release or for site-specific drug delivery. They are extensively used to design therapeutic systems, modify drug release, or mask unpleasant drug taste. Cellulose derivatives are characterized by different physicochemical properties, such as swellability, viscosity, biodegradability, pH dependency, or mucoadhesion, which determine their use in industry. One cellulose derivative with widespread application is ethylcellulose. Ethylcellulose is used in pharmaceutical technology as a coating agent, flavoring fixative, binder, filler, film-former, drug carrier, or stabilizer. The aim of this article is to provide a broad overview of ethylcellulose utilization for pharmaceutical purposes, with particular emphasis on its multidirectional role in the development of oral and topical drug dosage forms.

## 1. Introduction

Modern pharmaceutical technology could not exist without polymers, which play an integral role in the advancement of drug delivery (e.g., by providing controlled release of therapeutic agents, masking bitter taste of drugs, or serving as carriers in targeted therapy). They have a wide range of physicochemical characteristics according to their molecular weight and configuration. Therefore, polymers approved for use in medicines are receiving considerable attention as essential excipients in the development of different drug dosage forms. Cellulose derivatives are an important group among the most commonly used polymers in pharmaceutical technology. Cellulose is one of the most abundant polymers in nature. It is produced by photosynthesis and constitutes a basic plant component. It is a linear polymer composed of glucopyranose residues, where the units are connected by 1,4-β-glycosidic bonds. It is a tasteless and odorless component with fibrous structure that is practically insoluble both in cold and hot water. Among the cellulose modifiers, its esters and ethers are of practical importance. Cellulose-based biomaterials are widely utilized as biocompatible templates for designing novel drug delivery systems with a wide range of pharmaceutical applications via different routes and pharmacotherapeutic purposes [1,2,3,4].

Ethylcellulose (EC), an ethyl ether of cellulose, is a free-flowing, white to light-tan powder prepared from wood pulp or cotton that is used in pharmaceutical manufacturing industries. The European Pharmacopoeia (Ph. Eur.) and United States Pharmacopoeia (USP) monographs describe EC as partially O-ethylated cellulose [5,6,7]. EC with ethoxyl substitution molecular formula is C_12_H_23_O_6_(C_12_H_22_O_5_)*n*C_12_H_23_O_5,_ where *n* can vary in order to provide a wide variety of molecular weights, which affect its properties. It is insoluble at any pH that occurs in organisms, but it undergoes swelling in the presence of gastric juice [3,4]. There is very limited data detailing possible side effects of EC, hence it is generally regarded as safe (GRAS) and included in the US Food and Drug Administration (FDA) Inactive Ingredients database as well as in the Canadian List of Acceptable Non-medicinal Ingredients to be utilized in oral capsules, suspensions, tablets, topical emulsions, and vaginal or ocular preparations. In contrast to other polymers which are insoluble in the gastrointestinal tract (e.g., nitrocellulose, cellulose acetate, Eudragit RL, or Eudragit RS), EC is characterized as a non-ionic material, having the advantage of being non-reactive. Moreover, safety data on utilizing cellulose acetate and methacrylic acid copolymers in pediatric preparations are limited, while EC is clinically tested and approved for use in pediatric formulations. EC is also allowed for use in non-parenteral medicines licensed in Europe. The polymer is accepted for use as a food additive because it is a non-calorific and metabolically inert substance following oral consumption. As EC is not considered to be a health hazard, the World Health Organisation (WHO) has not specified its acceptable daily intake [4,8,9]. According to the intended use, the commercially available EC can be classified in two categories of products: industrial grade and pharmaceutical grade. For the pharmaceutical grade, the quality standard should meet USP, Ph. Eur., Chinese Pharmacopoeia, and Japanese Pharmacopoeia standards [6,7,10,11].

## 2. Physicochemical Characteristics of EC

**Figure 1:**
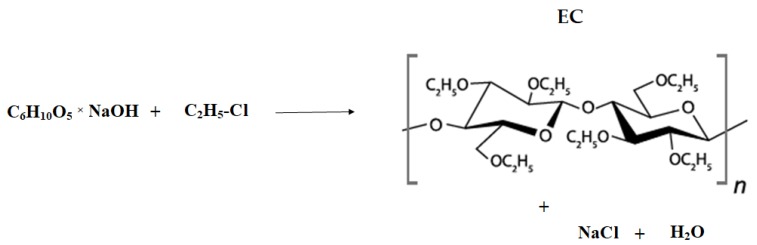
Schematic illustration of ethylcellulose (EC) obtainment.

The polymer is obtained by synthesis (etherification) through the substitution of the cellulose hydroxyl groups with ethoxyl ones. The chemical reaction involves cellulose dissolution in sodium hydroxide aqueous solution, which leads to degradation of the cellulose’s molecular structure. This results in the formation of an alkali cellulose and exposure of the cellulose hydroxyl group for reaction. Afterwards, ethyl chloride gas is added to the reaction medium, leading to bonding with the alkalized cellulose. EC, sodium chloride, and water are formed (Figure 1) [1,2,3,4,5].

EC contains 44–51% ethoxyl groups (-OC_2_H_5_) and is composed of β-anhydroglucose units joined together via acetal linkage. EC is a biocompatible, non-allergenic, nonirritant, colorless, odorless, and tasteless hydrophobic polymer soluble in a wide variety of organic solvents (alcohols, ketones, and polycyclic aromatic hydrocarbons), but does not dissolve in water, glycerin, or propylene glycol. EC that contains no less than 46.5% of ethoxyl groups is freely soluble in chloroform, ethanol, ethyl acetate, methanol, and toluene. EC is compatible with a wide array of excipients and most of the plasticizers used in pharmaceutical formulations, and therefore can be well mixed with many softeners, oils, and waxes (dibutyl phthalate, diethyl phthalate, dibutyl sebacate, triethyl citrate, tributyl citrate, acetylated monoglyceride, butyl and glycol esters of fatty acids, refined mineral oils, oleic acid, stearic acid, stearyl alcohol, castor oil). It shows incompatibilities with paraffin and microcrystalline wax. EC exhibits a high degree of stability within pH 3–11, making it useful both in acidic and alkaline mixtures. EC is also slightly hygroscopic, absorbing very little water from humid air or during immersion. In addition, it can form tough and tensile films and maintains flexibility even at low temperatures. It possesses good thermal stability and low ash content when burning [1,3,4,5].

The physical characteristic of EC types and their performance depends on the degree of etherification or substitution (ethoxyl content), molecular uniformity, and molecular weight of the cellulosic backbone. Solubility in organic solvents is achieved with degree of substitution (DS) values between 2.2 and 2.6. A range of commercial products is available with a degree of substitution between 2.15 and 2.60, corresponding to a range of ethoxyl contents from 43% to 50%. At low DS values (0.8–1.3), the replacement of some of the hydroxyl groups by ethoxyl groups reduces the hydrogen bonding across the cellulosic chains to such an extent that the material is soluble in water. Further replacement of hydroxyl groups by the less polar and more hydrocarbon ethoxyl groups increases the water resistance. Fully etherified EC is soluble only in non-polar solvents [1,2,3,4,5].

The molecular weight can be regulated by controlled degradation of the alkali cellulose in the presence of air. This can be done either before or during etherification. The molecular weight of commercial grades is usually expressed indirectly as viscosity. The completely etherified material with a degree of substitution of 3 has an ethoxyl content of 54.88%. The viscosities are determined using a capillary viscometer and recorded in millipascal-seconds (mPa·s) or centipoise (cP) synonymous for a 5% *w/w* solution in toluene/ethanol solvent mixture (80:20), being determined at 25 °C and expressed in mPa·s. For each viscosity grade of EC, the pharmacopoeial specification allows variation of 80–120% within the stated nominal viscosity (depending on manufacturer). Several types of EC (e.g., Ethocel^™^ 4, Ethocel^™^ 10, and Ethocel^™^ 45) differ in the length of the polymer chains, the rate of dissolution, and the viscosity of their solutions. The mechanical properties are largely determined by chain length; softening point, hardness, water absorption, and solubility are rather more influenced by the degree of substitution [1,2,3,4,5,12,13].

EC viscosities increase with the increase in the polymeric backbone chain length. The impact of EC molecular weight variation (measured by viscosity) on the drug release was determined by Mehta et al. Metoprolol was selected as a model drug that is highly soluble in water and acetaminophen as one with low aqueous solubility. Drug-layered sugar spheres were coated with EC of different viscosity grades at varying coating weight gains, and their drug release profiles were determined. The study revealed that viscosity variation within the manufacturer’s specifications of EC (Ethocel^™^ 10, 20, and 100 cP) had a minimal effect on release of model drugs. Acetaminophen multiparticulates exhibited slower drug release and longer lag time when compared to the metoprolol beads. The obtained results can be associated with the lower aqueous solubility of acetaminophen compared to metoprolol. Based on the study, it was concluded that all grades of EC were suitable for organic solvent coating of extended release barrier membrane multiparticulates [14]. In another study, sustained-release metformin hydrochloride nanoparticulate systems were developed, and the effect of different viscosity EC grades on their in vitro characteristics was investigated. The sustainability of EC was enhanced by the increase in its apparent viscosity—the EC with higher viscosity grade sustained metformin release more efficiently [15].

## 3. Pharmaceutical Characteristics of EC

EC has been widely used in the pharmaceutical industry for decades, being utilized in oral and topical pharmaceutical formulations for various purposes. It has the potential to modulate and improve the physiological performance of drug dosage forms because of its hydrophobic nature and swelling capacity. The main goal of EC utilization is the development of drug dosage forms with modified release (MR), as EC ensures drug dissolution in the entire gastrointestinal tract, providing constant drug concentration and eliminating the necessity of taking several doses a day, hence improving pharmacotherapeutic effectiveness. Because it is an inert hydrophobic polymer and possesses properties such as lack of toxicity, stability during storage, and good compressibility, it is suitable for designing sustained-release preparations [1,2,3,4,5,16]. EC provides the formation of hydrophobic coatings, filaments, or backing layers; masks unpleasant medicine tastes; creates matrices and nanostructures for the preparation of bioactive materials or is used as an encapsulation excipient for the preparation of sustained-release microparticles; and serves as moisture protector or binder. It can also be used as a dispersing, stabilizing, and water-retaining agent to prevent drugs from getting wet and to promote the safe storage of drugs. Shell tablets can be obtained by suspending drug in the gastrointestinally insoluble carrier. EC has also been used as a matrix in the preparation of both water-soluble and sparingly water-soluble drugs using solid dispersion technique. It is ubiquitously utilized as a coating material in sustained-release preparations due to its film-forming properties and good mechanical strength. Also, in the coating context, its important feature is insolubility at any physiological pH. However, the polymer exhibits swelling in the presence of gastric juice, making it permeable for water and permitting extended modified drug release [4,17,18,19,20,21,22]. EC-coated beads have also demonstrated the ability to absorb pressure and hence protect the coating from fracture during compression. Drug release from EC-based film coatings depends on the coating level, drug solubility, and the form in which the polymer is applied in the coating process (e.g., as powder, aqueous dispersion, or organic solution). It is also common to employ blending polymers to get suitable and desired results, as using a single polymer may not give the desired drug release profile. Owing to its hydrophobic properties, EC reduces the penetration of water into the solid polymeric matrix, hence reducing drug release [4,23].

**Figure 2:**
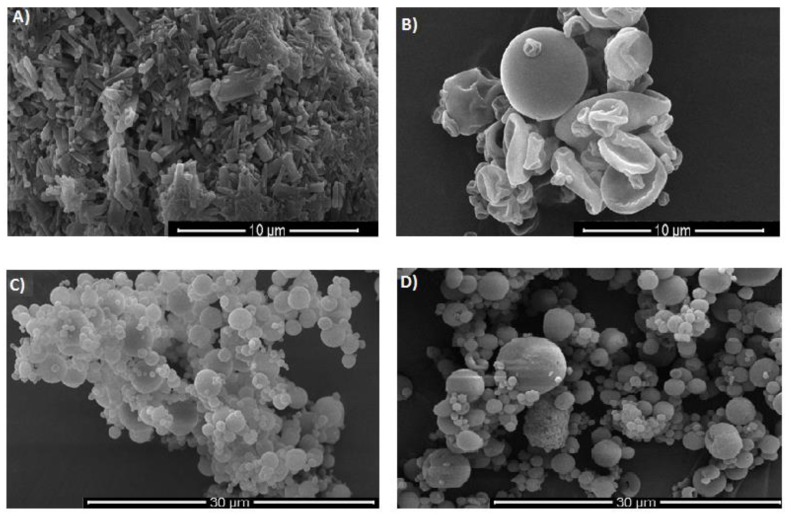
SEM pictures of (**A**) EC in powder form; (**B**) spray-dried ethanolic solution of EC (inlet temperature 65 °C, aspirator flow 98%, feed flow 3.5 mL/min); (**C**) spray-dried Surelease^®^; and (**D**) spray-dried Aquacoat™ ECD-30 (author’s pictures under magnification 10,000× (**A**,**B**); 5000× (**C**,**D**)).

EC can be found in different forms, such as powders with various viscosity grades or aqueous dispersions. Examples of organic solids are Ethocel^™^ or Aqualon^™^, and aqueous dispersions include Surelease^®^, Aquacoat^®^ ECD, Aquarius^™^ Control ECD, and AshaKote^®^ (Table 1, Figure 2) [12,24,25,26,27,28]. Commercially available types of EC (e.g., Ethocel^™^ 4, Ethocel^™^ 10, and Ethocel^™^ 45) differ in the length of the polymer chains, the rate of dissolution, molecular weights, and hence the viscosity of their solutions (ranging from 3 to 110 mPa·s) [12].

## 4. Applicability of EC in Pharmaceutical Formulations

### 4.1. EC as An MR Coating Material in Oral Delivery Systems

**Figure 3:**
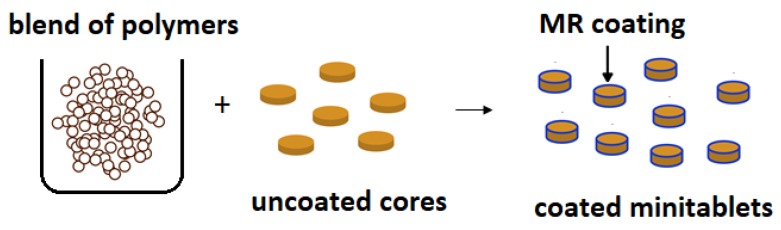
Schematic illustration of coated modified release (MR) minitablets.

Because of EC’s potential to modify drug release, it is widely studied as a coating agent. EC as release retardant was employed in a once-a-day sustained-release system of tacrolimus. The pellets were coated with EC using a fluid bed granulator. Drug release was markedly impeded by the outer EC-based coating layer, displaying about 60% drug release after 8 h, regardless of the acidity of the medium. It was assessed that there were no statistical differences between the obtained pellets and the marketed sustained-release capsules (Advagraf^®^) [29,30]. Shah et al. developed a multiunit formulation for the colon-targeted delivery of metronidazole using EC and Eudragit^®^ S 100 as coating polymers to prevent initial drug release in the gastric region (Figure 3). Cores of mini tablets containing drug were prepared using suitable swelling agents to provide pH-sensitive pulsatile drug delivery [31].

A double-layer coated colon-specific drug delivery system was developed by Kim et al. The system consisted of a chitosan-based polymeric subcoating of the core tablet (containing citric acid for microclimate acidification), followed by an enteric EC coating. The system showed drug release in a controlled manner by inhibiting drug release in the stomach and intestine, but releasing the drug gradually in the colon [32]. The example of utilizing EC as a release-retarding coating is a commercially available preparation called Micro-K^®^ in hard gelatin capsules, containing small crystalline particles of potassium chloride (KCl). Each crystal of KCl is microencapsulated by a patented process with an insoluble EC polymeric coating which functions as a semi-permeable membrane ensuring controlled release of K^+^ and Cl^−^ ions. Fluids pass through the membranes and gradually dissolve KCl within the microcapsules. As a result, drug slowly diffuses outward through the membrane over an 8–10 h period [33,34]. Another example is Theo-24^®^—the first commercial product for 24-h theophylline therapy launched on the market. The technology utilized in the formulation uses a chemical timing complex (protected by patent) to produce very small theophylline-coated beads that provide dependable zero-order controlled drug release. Tiny spheres of a sugar and starch blend form the core of the beads. The core is first coated with theophylline and then with a timing complex utilizing EC. The resulting beads are put into capsules. When the capsule dissolves in the gastrointestinal tract, the insoluble coating on the bead slowly erodes. The drug, which is highly soluble, moves through the coating. In the core, the starch swells and pushes the drug out while the dissolving sugar also helps carry the drug through the chemical timing complex, which results in a constant release [33,35].

Matrix tablets of hydrochlorothiazide were coated with an insoluble barrier membrane using aqueous EC coating (Surelease^®^) and HPMC-based Opadry^®^ as a pore former, at 85:15 *w/w* ratio. The combination of barrier membrane and hydrophilic matrix system was utilized as a strategy to modulate drug diffusion from hydrophilic matrices and to reduce the overall variability in release [36]. The aims of another study were to control the release of a water-soluble theophylline from mini matrices made of HPMC by applying an EC film coating (Surelease^®^) and to assess coating load on release rates. At low coating weight gains, tablets released the entire drug within 0.5 h, while at high coating weight gains only a very small amount (<5%) of drug was released after 12 h [17].

**Figure 4:**
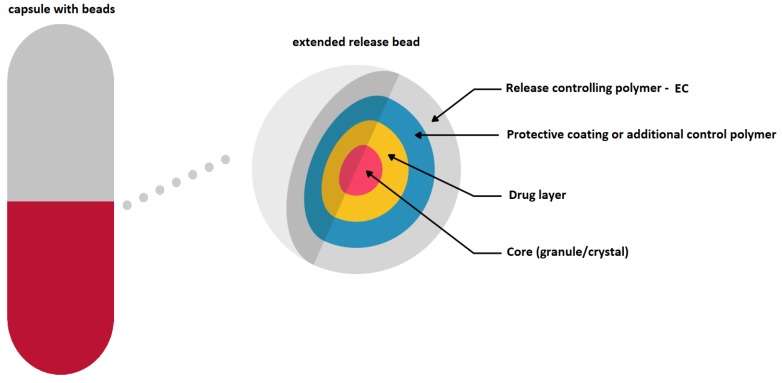
Schematic illustration of Diffucaps system in a Metadate CD^®^ capsule. Reprinted with permission from Reference [16]. Copyright 2019 MDPI.

EC has found its application as a coating material in commercially available modern MR solid dosage forms (Diffucaps, DiffCORE^™^, SODAS^®^, or Geomatrix^®^ systems). It is also used in medicines dedicated to patients suffering from ADHD: Adzenys XR^®^-ODT and Cotempla^®^ XR-ODT [16,33,37,38,39,40,41,42]. Diffucaps are a multiparticulate system, where drug profiles are created by layering a drug onto a neutral core (e.g., sugar spheres, crystals, or granules) followed by the application of a rate-controlling functional EC membrane. Such a system is applied in Metadate CD^®^ capsules (Figure 4) [16,37].

**Figure 5:**
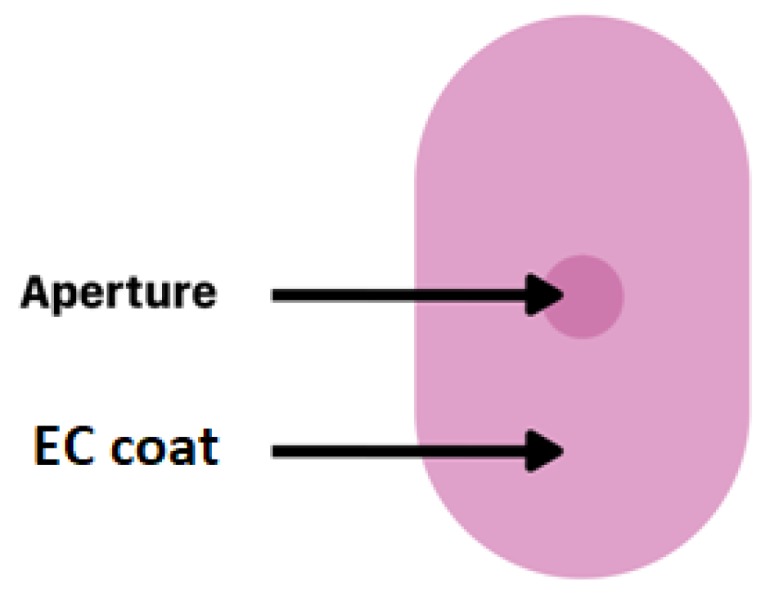
Schematic illustration of DiffCORE^™^ system. Reprinted with permission from Reference [16]. Copyright 2019 MDPI.

Another example of an EC-coating-based oral system is DiffCORE^™^ (Lamictal^®^ XR) (Figure 5). DiffCORE^™^ is a technology developed to achieve extended release by delivering drug from a tablet core through one or several apertures in an impermeable coat made of EC. The technology combines the use of apertures that are mechanically drilled into functional film-coated tablets (on both faces of the tablet’s structure) with a polymer coating that controls the mechanism of diffusion. This combination is designed to ensure the dissolution rate of drug over a period of approximately 12–15 h [16,38].

**Figure 6:**
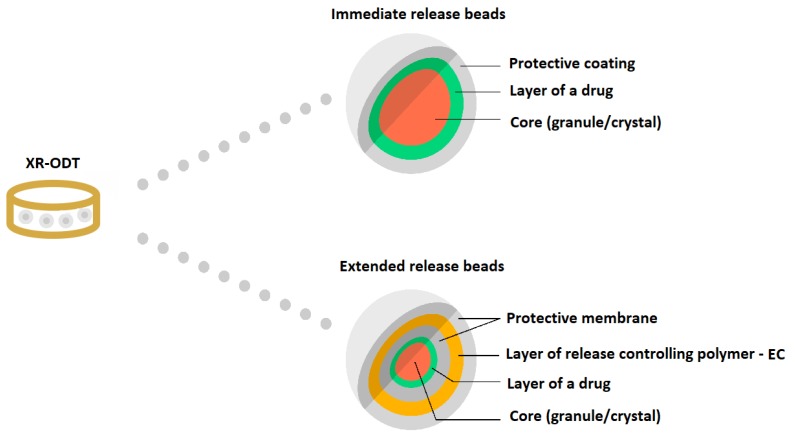
Schematic illustration of XR-ODT system. Reprinted with permission from Reference [16]. Copyright 2019 MDPI.

Adzenys^®^ XR-ODT and Cotempla^®^ XR-ODT are examples of orally disintegrating tablets which dissolve quickly in the mouth, maintaining extended drug release along gastrointestinal tract. The technology utilized in the medicines uses two different types of particles (in different ratios depending on the formulation): immediate release and extended release. Two different polymer coatings are applied to the extended release beads: interior polymer coating as diffusion barrier (EC) and pH-dependent exterior polymer coating (methacrylic acid) (Figure 6) [16,41,42].

**Figure 7:**
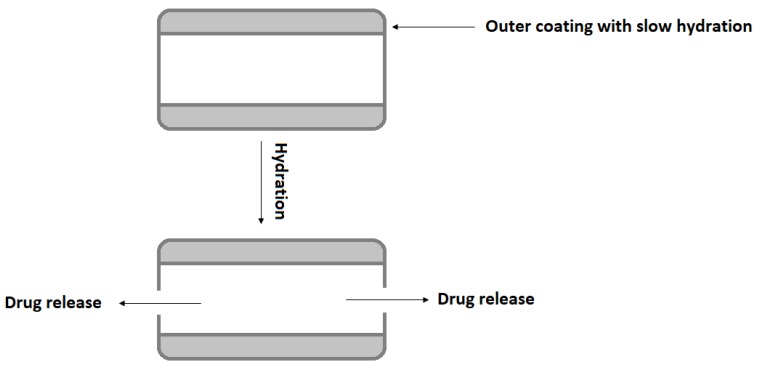
Schematic illustration of drug release from Geomatrix^™^ system modified from Wan et al. [33].

Inderal^®^ LA, long-acting capsules with propranolol, uses polymer-coated controlled diffusion technology to achieve 12 h release of drug for hypertension treatment. Inderal^®^ LA consists of small spheroids placed in a gelatin capsule. Each spheroid containing propranolol and a microcrystalline cellulose is coated with a porous membrane made of a mixture of EC, HPMC, and plasticizer [33,43]. Dilacor XR^®^ is a one-per-day oral formulation of diltiazem provided in capsule. The capsules contain degradable, controlled-release tablets designed based on Geomatrix^®^ technology to release diltiazem over a 24 h period. Geomatrix^™^ is a patented controlled-release system incorporated in the tablets. Each capsule contains multiple extended-release diltiazem tablets, consisting of two inactive surfaces sandwiched around the drug core (made of HPMC and hydrogenated castor oil) swellable in an aqueous medium. The inactive surfaces are composed of a methylcellulose–EC combination. The drug is released as a result of the swelling of the core, which acts as a drug reservoir. Controlled release of diltiazem begins within 1 h, with maximum plasma concentrations being achieved 4–6 h after administration. The inactive surfaces hydrate at a rate much slower than the core, thus regulating the drug release and assuring constant 24 h medicine delivery (Figure 7) [33,44].

**Figure 8:**
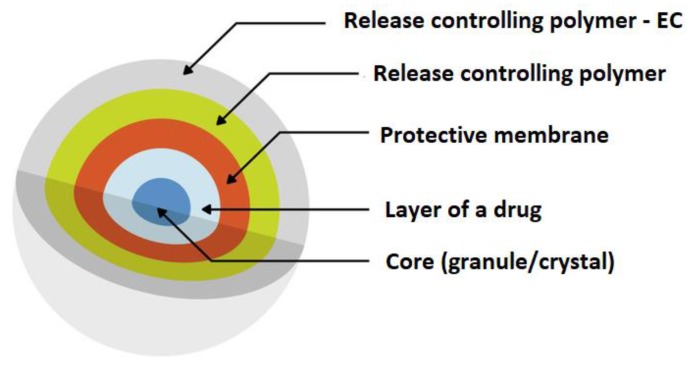
Scheme illustration of SODAS^®^ delivery system modified from Elan drug technologies [39]. Reprinted with permission from Reference [16]. Copyright 2019 MDPI.

An interesting example of applying EC as a coating is SODAS^®^ (Spheroidal Oral Drug Absorption System), utilized in Cardizem^®^ CD. The medicine is formulated as capsules and consists of two populations of sustained-release beads that differ only by the thickness of the polymer (EC) surrounding them. The EC membrane contains added water-soluble polymers which dissolve and create pores in the membrane. The polymer beads release 40% of the total diltiazem amount in the first 12 h (Figure 8) [33,39,40,45].

Core elements of drugs coated with a water-insoluble polymer such as EC offer reduced dissolution profiles, and as a consequence provide a taste-masking effect [46,47]. EC was used as taste-masking agent for quinine, utilizing a spray drying method. The obtained results showed that the quinine dissolution rate was altered, the bitter taste of quinine was successfully masked, and its intestinal absorption was simultaneously controlled [48]. EC for taste-masking purposes was tested in organic (Ethocel^™^) and aqueous form (Surelease^®^). To determine whether the designed particles effectively masked the bitter taste of rupatadine fumarate, three independent methods were used: a human taste panel, an in vitro release test conducted in conditions mimicking the oral cavity environment, and an electronic taste sensing system (electronic tongue). It was clearly confirmed that particles formulated from aqueous dispersions of EC provided a very effective taste-masking barrier [49]. Evaluation of the use of Surelease^®^ as a barrier membrane coating with pediatric precedence of use on the taste-masking of immediate-release acetaminophen granules was also conducted. The dissolution profiles of obtained granules were successfully modified using a Surelease^®^ and Opadry^®^ blend as a barrier membrane, providing slow initial drug release [50]. Another study concerned the development of orally disintegrating caffeine citrate tablets utilizing hot melt extrusion technology (EC was used as a polymeric taste-masking carrier) [51]. EC was also applied as a taste-masking and release-slowing agent to develop a gabapentin nanosponge-based controlled release dry suspension for pediatric use, using the suspension layering technique [52].

Utilizing of EC as release modifier in marketed oral formulations is presented in Table 2.

### 4.2. EC as a Sustained Release Material in Topical Delivery Systems

The utilization of EC to obtain a sustained release profile is also exploited in preparations for external use (e.g., transungual, ocular, vaginal, or transdermal). Successful topical therapy depends on effective drug release and penetration into the objective area, which can be achieved using an adequately developed drug dosage form [1,2,3,4,5,65]. EC can be applied in transungual delivery systems as a release modifier and a biocompatible agent ensuring optimal viscosity of the formulation. An isotretinoin nail lacquer intended for the treatment of nail psoriasis was developed, and its penetration efficiency across the nail plate was accessed. Lacquer with EC (at 6% concentration) possessed improved handling characteristics and enhanced drug distribution across the nail [66]. Another study documented an antifungal nail lacquer with miconazole developed with 0.5% EC which extended drug release up to 48 h [67]. Šveikauskaitė et al. studied naftifine hydrochloride release from experimental nail lacquer formulations obtained from EC and Eudragit. By using microcalorimetry they revealed possible interactions between naftifine and EC [68].

Eye administration is a demanding issue in pharmaceutical technology, and to enhance ocular bioavailability, sustained-release drug dosage forms such as hydrogels, inserts, contact lenses, or minitablets are designed. Among the materials employed in developing ophthalmic formulations, EC can be utilized as a polymer extending drug release [69] (Table 3).

Reports have also documented the application of EC as matrix material for transdermal patches for systemic delivery, showing desired permeation enhancement and flexibility [1,2,3,4,5]. Interestingly, EC has also been indicated in the formulation of a transdermal spray with clotrimazole to improve drug transport through the skin up to 12 h and to promote its in vivo antifungal activity [70].

Table 3 presents literature examples of EC utilization in ocular and transdermal drug delivery systems.

### 4.3. EC-Based Solid Dispersions

In preparation of MR dosage forms, the concepts of solid dispersions and drug incorporation in an insoluble EC carrier have been also explored [81,82,83,84,85]. Tsunashima et al. aimed to prepare MR formulation of tacrolimus to achieve both an extended release profile and improved drug solubility. Extended release solid dispersions of tacrolimus were successfully prepared via the solvent evaporation method using EC and HPMC as polymeric materials [82]. Sadeghi et al. compared characteristics of EC matrices prepared from solid dispersion systems with those prepared from a physical mixture of drug and polymer. Sodium diclofenac was used as a model drug. It was shown that matrices prepared from physical mixtures were harder than those prepared from solid dispersion systems, and their release rates were considerably faster. In the solid dispersion system, drug particles were incorporated in an EC matrix, which caused a great delay in drug diffusion through the polymer and made diffusion a rate-retarding process in the drug release mechanism [83]. Evaluation of the release mechanism of a poorly water-soluble drug (indomethacin) from extended-release solid dispersion systems with EC and HPMC was performed by Ohara et al. The dissolution behavior of indomethacin depended on the structures of EC–HPMC matrices and showed pH dependency—the drug dissolution rate was slower in an acidic environment than in a neutral one. The obtained results revealed that hydrophobic interaction between indomethacin and EC occurred under lower pH and strongly delayed the dissolution rate of the drug [84]. Sustained-release solid dispersions with EC and Eudragit RSPO were designed to control the release of verapamil hydrochloride. Solid dispersions obtained by a simple solvent evaporation technique and physical mixture formulations were compressed to tablets. The in vitro drug release study revealed that solid dispersions containing a combination of EC and Eudragit RSPO extended the release rate for 20 h compared to the physical mixtures at the same ratio, and that the release of verapamil from tablets containing solid dispersion could be effectively controlled [85].

### 4.4. EC-Based Micro- and Nanocarriers 

**Figure 9:**
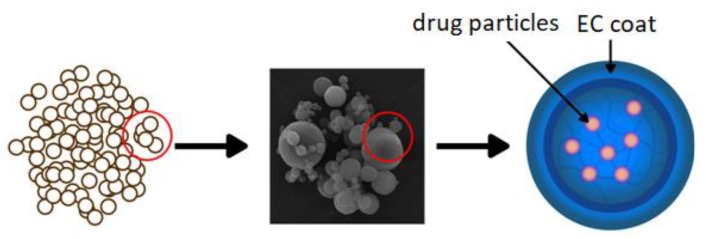
Schematic illustration of microparticles as a drug delivery system. Reprinted with permission from Reference [16]. Copyright 2019 MDPI.

A common approach to modifying drug release is preparing polymeric micro- or nanocarriers which contain drug enclosed in the polymer shell or incorporated in the polymer matrix (Figure 9). Micro- and nanoparticles have gained significant interest for their use in various drug formulations not only to sustain drug release, but also in order to improve bioavailability, decrease side effects, or increase drug stability [86]. An extended-release oral flexible tablet (ER-OFT) formulation was developed using carbamazepine as model drug for pediatric and geriatric compliance. Microparticles of carbamazepine were prepared using EC as a matrix polymer and HPMC as a hydrophilic pore former utilizing a high-shear granulator fitted with an atomizing spray system. Granulation of carbamazepine and EC with ethanolic binder solution resulted in obtaining microparticles with 16 h extended release [87]. EC microparticles can be formulated using a variety of techniques: interfacial and in situ polymerization, coacervation phase separation, spray drying, spray congealing, or by rotary fluidization bed granulator method [88].

As the extreme acidic environment of the stomach contributes to poor success in the treatment of *Helicobacter pylori* infections, Pan-In et al. encapsulated clarithromycin into particles made of pH-resistant polymer (i.e., EC). Nanoparticles were prepared via a simple anti-solvent particle induction method [89]. Similarly, EC nanocomposites containing rifampicin obtained by supercritical anti-solvent process prolonged drug release and increased its bioavailability [90]. Interestingly, EC was also tested as a carrier for protecting the L-alanyl-L-glutamine peptide and simultaneously providing its prolonged release [91].

Shankar et al. formulated EC microparticles for the vaginal delivery of metronidazole by thermal change method [92]. EC was also utilized in the development of nanoparticles for the topical delivery of corticosteroids [93] or to modulate the release and reduce the ulcerogenicity of piroxicam after oral administration [93]. A reduction of 66% in mean ulcer index was observed, indicating that the obtained particles had a significant potential of offsetting deleterious side effects common in piroxicam use [94]. Similarly, as etoricoxib has many side effects when taken orally, EC nanoparticles produced by a nanoprecipitation technique were designed. Etoricoxib-loaded EC nanoparticles for local drug delivery in arthritis provided sustained drug release, thereby improving patient compliance [95]. Interestingly, nanocapsules of beclomethasone were prepared for pulmonary delivery using EC as a protective polymer. They were characterized by delayed drug photodegradation, prolonged drug release without burst effect, and insignificant cytotoxic effect [96].

EC can also be utilized to prepare sustained-release oral liquids, such as syrup with hydrocodone and chlorpheniramine (Tussionex^®^) or suspension with mirabegron based on ion-exchange technology [97,98,99].

Maulavi et al. tested EC-based-microparticles-laden hydrogel contact lenses to provide the sustained ocular delivery of timolol. Prototype poly(hydroxyethyl methacrylate) hydrogel contact lenses improved drug bioavailability due to the increase in ocular residence time [100]. Timolol-encapsulated EC nanoparticles lenses for controlled sustained ocular drug delivery were also evaluated in vivo using rabbits. Nanoparticles were prepared by a double emulsion method and incorporated into acrylate hydrogel, then implanted in hydrogel contact lenses. They exhibited extended timolol release and an intra-ocular pressure lowering effect for 192 h in rabbits without significant ocular complications [101].

Application of EC microparticles containing naproxen to textile materials was studied by Arici et al. Microparticles were prepared by spray-drying using an aqueous EC dispersion and applied to orthopedic support materials. Since these materials are used for traumatic irritations, over-forced ligaments and tendons, and specific problems of various joints, it was concluded that they can serve as potential drug delivery systems [102].

Another approach to obtaining microcarriers is microsponge development. Microsponges are novel drug delivery systems, which ensure effective sustained drug release. They possess unique properties including self-sterilizability, due to the minimal pore size which does not allow bacteria to enter and contaminate the formulation. Microsponges can entrap drugs up to three times their weight due to their very high porosity. EC is utilized as a foundation material for microsponges and for microsponge engineering due to its nonirritating, nontoxic, and nonallergic nature [103,104,105]. Bothiraja et al. focused on the development of an EC microsponge gel as a topical carrier for the controlled release and cutaneous drug deposition of eberconazole. The microsponges were prepared using the quasi emulsion solvent diffusion method. An in vivo skin deposition study demonstrated higher retention in the stratum corneum layer as compared with commercial cream with eberconazole [106]. Jelvehgari et al. developed a microsponge delivery system to facilitate the topical delivery of benzyl peroxide. As benzyl peroxide has many side effects when applied per se in gel form, such a delivery system to the skin could reduce those effects while reducing its percutaneous absorption [107]. EC was also utilized to prepare microsponges to overcome problems connected with the poor water solubility, low stability, and high volatility of tea tree oil. Microsponges loaded with tea tree oil were prepared using EC and polyvinyl alcohol and incorporated into carbopol gel [108]. Another study describes the development of the topical delivery of oxybenzone—one of the most widely used chemical filters found in commercial sunscreens—using a microsponge-loaded gel. As the ingredient can cause dermatitis and skin irritation, oxybenzone-loaded microsponges were successfully formulated by a quasi emulsion solvent diffusion method with EC utilization. It was shown that controlled release of oxybenzone from the microsponge structures and barrier effect of gel resulted in prolonged retention with reduced permeation, reduced irritation, and enhanced sun protection factor [109]. The aim of another study was to develop a sustained-release delivery system of tacrolimus, formulated using a microsponge base, employing EC and xanthan gum by modified double emulsification techniques [110]. EC microsponges can be designed for ocular delivery; acetazolamide formulated in a microsponges–Pluronic F-127 in situ gel was characterized by improved therapeutic efficacy and reduced systemic side effects of oral acetazolamide. The obtained formulation showed higher therapeutic efficacy compared to free drug in gel [111].

EC has also been studied as a material for nanofibers, which possess a broad range of new applications in pharmaceutical technology [112]. Over the past years, electrospinning has been attractive as a simple, reproducible, versatile, and cost-effective technique for nanofiber production. Electrospun nanofibers have been extensively used for different biomedical applications, including wound dressing, tissue engineering, and drug delivery. The fiber morphology can be manipulated by changing the solvent ratio, resulting in a decreased fiber diameter. Membranes made of EC nanofibers possess good physical properties and are characterized by beneficial air permeability [113,114]. Drug delivery rate is affected by the polymer type, fiber diameter, and drug concentration in the fiber [114,115]. EC as a filament-forming matrix was tested in ketoprofen nanofiber delivery systems designed by triaxial electrospinning [116]. EC was also utilized for creating a 5-fluorouracil loaded core for the fabrication of electrospun fibers [117] and in preparing water-stable composite nanofibers loaded with indomethacin with a sustained, diffusion-controlled release profile [118]. The inclusion of EC as a matrix former improved mechanical properties of the entire delivery system [118]. Another study employed blend fibers of poly(N-vinylcaprolactam) and EC with the aim of developing thermoresponsive sustained release formulations fabricated by twin-jet electrospinning containing ketoprofen. The obtained fibers were largely smooth and homogeneous, and the addition of a drug did not affect their morphology [115]. Additionally, Liu et al. reported that nanofibers obtained by electrospinning using blends of EC and gelatin were characterized by fine morphology and possessed improved thermal stability [119].

### 4.5. EC in Mucoadhesive Delivery Systems

In the development of dosage forms applied on mucous membranes, the selection of suitable polymers with adhesive properties is a crucial issue. Polymers that are bioadhesive and do not dissolve before releasing the incorporated drug are highly appreciated for sustain drug release. As a water-insoluble polymer, EC is often used as backing membrane for its film-forming property, low water permeability, drug impermeability, and moderate flexibility. It possesses bioadhesive properties, however lesser than Carbopol and chitosan [120]. Bagul et al. evaluated the in vitro mucoadhesive strength of various polymers and reported the following ascending order for force of adhesion expressed in Newtons (N): gelatin (1.42) < gum dammar (1.47) < gum copal (1.52) < ethyl cellulose (1.60) < sodium alginate (1.71) < xanthan gum (1.81) < chitosan (1.91) < HPMC (2.25) < carbopol (2.40) [120].

EC forms a hydrophobic network when the mixture comes into contact with water, resulting in sustained drug release. The utilization of EC as a single polymer or in conjunction with other adjuvants in the preparation of film-like drug carriers is well documented. Drug-loaded EC films are characterized by good adhesion, mechanical strength, and sustained release profile. They provide a flexible diffusion barrier and its properties can be changed by the amount of pore-forming agent, film thickness, and EC molecular weight [1,3,4,5]. Abruzzo et al. designed buccal films for propranolol administration. A polymeric layer was prepared by casting and drying with polyvinylpyrrolidone or polyvinylalcohol and the addition of gelatin or chitosan. EC was employed to formulate a second layer was applied onto the primary one in order to obtain prolonged drug delivery, increase adhesion, and mask the drug’s bitter taste [121]. A bilayer mucoadhesive buccal film containing a combination of ornidazole and dexamethasone was prepared using solvent casting to treat oral ulcers, with EC utilized as a backing layer. The formulation showed favorable swelling characteristics, and both drugs were released at 95% after 4 h [122]. EC was also applied for the preparation of allantoin-loaded films for the management of dry mouth syndrome. The findings revealed that the produced films were functional, mucoadhesive, flexible, and stable, with the potential for treating various intraoral diseases [123]. EC-based mucoadhesive buccal films containing fluticasone formulated by solvent casting technique showed acceptable physicochemical properties, homogenous drug distribution, adequate mucoadhesion time, moderate swelling, and sustained drug release up to 12 h [124]. The objective of another study was to develop a two-layered buccal mucoadhesive system consisting of a highly water-soluble drug (i.e., risedronate). Varied concentrations of chitosan, HPMC, and EC acting as an impermeable backing membrane ensuring sustained release were tested. The obtained systems showed good swelling and mucoadhesive characteristics, with 90–100% drug release within 8–12 h [125]. EC, as a biocompatible backing layer, was also utilized in the development of mucoadhesive bi-layered strips used in dental treatment for the controlled delivery of lidocaine [126]. EC dissolved in N-methyl pyrrolidone was successfully used as a polymeric matrix for the in situ forming gel (with doxycycline, metronidazole, and benzyl peroxide) as dosage form applied in periodontal pocket in periodontitis treatment. It turned out that increasing the amount of EC increased the viscosity of system while still exhibiting Newtonian flow and simultaneously decreasing the release of drug [127]. Another prolonged-release mucoadhesive gel containing metronidazole for periodontal application was developed basing on a mixture of glycerylmonooleate and EC. EC reduced the initial metronidazole release and significantly sustained its release profile [128]. Pathak et al. developed a mucoadhesive coating for fluconazole tablets for oral thrush treatment. EC coating reduced fluconazole permeation through the buccal mucosa, thereby ensuring high local drug concentration and faster provision of the fluconazole minimum inhibitory concentration in the oral cavity [129].

## 5. Conclusions

EC is a water-insoluble cellulose derivative with attractive features such as biocompatibility, gastroresistance, and degradation to non-toxic and readily excreted products. It is extensively utilized in pharmaceutical technology. It is generally recognized as safe to use, even in pediatric therapy. EC is easy to process alone or with the addition of other excipients (e.g., plasticizers or other polymers). Considering its multifunctional properties, EC is being widely exploited in oral and topical drug dosage forms. EC’s main advantage is its ability to modify the release of the drug, which allows for the creation of controlled delivery systems—the unique tailored carriers for many pharmaceuticals. EC-based materials are widely utilized as matrices for designing novel drug delivery systems with a wide range of applications.

## Figures and Tables

**Table 1:** Characteristics of commercially available EC *.

**Organic EC products**
**Ethocel^™^**
Available in many varieties that differ in grade and viscosity (e.g., Ethocel^™^ Standard Grade 4 Premium, 7 Premium, 10 Premium, 20 Premium, 45 Premium, 100 Premium). Ethocel^™^ are white to light-tan granular powders in physical appearance with bulk density and specific gravity of about 0.4 g/cm^3^ and 1.12–1.15 g/cm^3^ respectively and glass transition temperatures range between 129 and 133 °C. They dissolve in a wide range of solvents such as aliphatic alcohols, chlorinated solvents, and natural oils. They are practically insoluble in glycerin, propylene glycol, and water. Films made from Ethocel^™^ are tough, with high tensile strength and high flexibility even at low temperatures. They can be combined with water soluble polymers such as methylcellulose and hypromellose (HPMC) in aqueous coating liquids. They are characterized by thermoplastic nature and ability to soften at 135–160 °C which makes them versatile in pharmaceutical hot melt extrusion processes. They ensure desired drug release properties and improved bioavailability of especially poorly soluble drugs [12].
**Aqualon^™^ ethylcellulose**
During preparation, the substitution of ethoxyl groups is controlled to obtain commercially useful range of 48–52% ethoxyl (or 2.3–2.6 ethoxyl groups out of a theoretical maximum of 3.0) per anhydroglucose unit. Over this ethoxyl range, Aqualon^™^ ethylcellulose is classified into three ethoxyl types: N (low substitution), T (mid substitution), and X (high substitution). The improved compressible grade (Aqualon^™^ T10) was developed with optimized compactability (high ethoxyl content and low viscosity) and good powder flow. The grades of Aqualon^™^ ethylcellulose are compliant with the monograph requirements of the United States Pharmacopoeia (USP) and the European Pharmacopoeia (Ph. Eur.) [24].
**Aqueous dispersion of ethylcellulose**
**Surelease^®^**
Surelease^®^ is a family of fully formulated, aqueous dispersion products which constitute plasticized aqueous dispersions of EC with 25% (*w/w*) solid content available in four types: E-7-19029, E-7-19030, E7-19040, E-7-19050. They are plasticized (depending on type) with dibutyl sebacate (3.5%-E-7-19029, E-7-19030) and oleic acid (1.9%-E7-19040). Surelease^®^ is produced in the following steps: EC is blended with plasticizer, then extruded and melted. The molten plasticized EC is then directly emulsified in ammoniated water in a high-shear mixing device under pressure. Ammonium oleate is formed in situ in order to stabilize and form the dispersion of plasticized EC particles. Then, purified water is added to achieve the final solids content. Applications of Surelease^®^ include beads and particles coating, matrix granulation (the dispersion can be used as a binder in wet granulation for the production of free-flowing granules, which can subsequently be compressed into tablets), taste-masking coating, or nutritional enteric coating [25].
**Aquacoat^®^ ECD**
It contains primarily EC with a surfactant and a stabilizer from the emulsion stage (sodium lauryl sulfate (SLS) and cetyl alcohol (CA)). Depending on type it consists of EC (24.5–29.5%), CA (1.7–3.3%), and SLS (0.9–1.7%). Traces of dimethylopolysiloxane to enhance foaming during distillation may also be present. EC is dissolved in a water-immiscible organic solvent with CA addition as a dispersion stabilizer. The solution is then emulsified into an aqueous SLS solution. The resulting crude emulsion is homogenized to yield a submicron emulsion which is then distilled to remove the organic solvent and water to yield a solid dispersion. EC is present in the dispersion as spherical particles in the size range of 0.1–0.3 μm. It exists as a milky white liquid with the characteristic odor of EC. Product does not contain plasticizer. Recommended plasticizers include dibutyl sebacate, acetylated monoglycerides, triacetin or glyceryl triacetate, acetyltriethyl citrate, and triethyl citrate. It is used for the aqueous film coating of solid dosage forms to extend drug release, taste mask, or to protect against moisture [26].

* The table presents examples of selected, most commonly used, products.

**Table 2:** Examples of commercially available oral solid dosage forms with EC as release modifier.

Product (Manufacturer)	Drug	Dosage Form	EC Role	Reference
Aciphex^®^ Sprinkle^™^ (Eisai Management Co., Ltd.)	rabeprazole	granules in capsule	coating, delayed release	[53]
Adzenys XR-ODT^™^ (Neos Therapeutics)	amphetamine	extended release orally disintegrating tablet with beads	coating, extended release	[41]
Advagraf XL^™^	tacrolimus	capsules	extended release	[30]
Cotempla XR-ODT^®^ (Neos Therapeutics)	methylphenidate	extended release orally disintegrating tablet with beads	coating, extended release	[42]
Cardizem CD^®^ (Abbot Laboratories)	diltiazem	tablets in capsule	coating, extended release	[45]
Dilacor XR^®^ (Actavis Pharma, Inc.)	diltiazem	tablets in capsule	extended release	[44]
Dilatrate-SR (Epic Pharma, LLC)	diltiazem	beads in capsule	extended release	[54]
Durlaza (New Haven Pharmaceuticals, Inc.)	acetylsalicylic acid	capsules	extended release	[55]
Enjuvia (Teva Pharmaceuticals USA, Inc.)	estrogens	coated tablets	extended release	[56]
Entocort EC (Astra Zeneca)	budesonide	coated granules in capsules	sustained release	[57]
Inderal^®^ LA (Wyeth Pharmaceuticals, Inc.)	propranolol	granules in capsules	coating, extended release	[43]
Levbid^®^ (Mylan Pharmaceuticals Inc.)	hyoscyamine	tablets	extended release	[58]
Metadate CD^®^ (UCB Manufacturing, Inc.)	methylphenidate	granules in capsule	coating, extended release	[37]
Micro-K^®^ (KV Pharmaceutical)	potassium	microcapsules in capsules	coating, extended release	[34]
Lamictal^®^ XR (GSK)	lamotrigine	tablets	coating, extended release	[38]
Orfiril Long (Desitin Arzneimittel GmbH)	natrii valproas	minitablets	extended release	[59]
Palladone (PF Laboratories Inc.)	hydromorphone	capsules	extended release	[60]
Pentasa^®^ (Ferring GmbH)	mesalamine	granules	prolonged release	[61]
Qsymia^®^ (Vivus, Inc.)	phentermine, topiramate	capsules	extended release	[62]
Tegretol^®^ XL (Novartis)	carbamazepine	tablets	matrix, extended release	[63]
Theo-24^®^ (UCB Pharma, Inc.)	theophylline	beads in capsule	coating, extended release	[35]
Trokendi XR^™^ (Catalent Pharma Solutions)	topiramate	capsules	extended release	[64]

**Table 3:** Examples of ocular and transdermal delivery systems based on EC.

Drug Dosage Form	Active Substance	Used Excipients	EC Role	Reference
**Ocular formulations**				
ocular inserts	brimonidine	sodium alginate, EC	hydrophobic, sustained release coating	[71]
ocular inserts	ciprofloxacin	gelatin, EC	rate-controlling, increasing residence time membrane	[72]
ocular insert	acyclovir	sodium alginate, EC	rate-controlling membrane	[73]
in situ ophthalmic hydrogel	besifloxacin	sodium alginate, xanthan gum, EC	increase the pre-corneal residence time	[74]
minitablet	ciprofloxacin	HPMC, sodium carboxymethyl cellulose, EC, hydroxyethyl cellulose, carbopol	sustained release	[75]
**Transdermal formulations**				
transdermal patch	amlodipine	EC	sustained release	[76]
transdermal patch	flurbiprofen	EC, propylene glycol, dibutyl phthalate	constant drug release	[77]
transdermal delivery system	topiramate	EC, povidone, Eudragit L 100, carbopol	extended release	[78]
transdermal patch	dexibuprofen	EC, povidone, di-N-butyl phthalate	matrix formation	[79]
transdermal patch	centchroman	EC, polydimethylsiloxane, propylene glycol, Di-n-butyl-phthalate	Film-forming polymer	[80]
